# Quality of life in rectal cancer patients after radical surgery: a survey of Chinese patients

**DOI:** 10.1186/1477-7819-12-161

**Published:** 2014-05-22

**Authors:** Xinxin Li, Xinming Song, Zhihui Chen, Mingzhe Li, Lifeng Lu, Ying Xu, Wenhua Zhan, Yulong He, Kaiwu Xu

**Affiliations:** 1 Department of Gastrointestinal Surgery, The First Affiliated Hospital of Sun Yat-sen University, No. 58 Zhongshan Er Road, Guangzhou 510080, China; 2 Institute of Medical Statistics and Epidemiology, Guangdong Pharmaceutical University, Guangzhou, China

**Keywords:** Health-related quality of life, Rectal cancer, Surgery, Chinese

## Abstract

**Background:**

We aimed to investigate the impact of sociodemographic and clinical characteristics on health-related quality of life (HRQoL) in disease-free survivors after radical surgery for rectal cancer in a Chinese mainland population.

**Methods:**

We performed a cross-sectional survey from August 2002 to February 2011 by use of the European Organization for Research and Treatment of Cancer (EORTC) QLQ-C30 and QLQ-CR38 questionnaires of 438 patients who underwent curative surgery for rectal cancer. Patients who were followed up for a minimum of 6 months, had no relevant major comorbidities and whose disease had not recurred were asked to complete both questionnaires. The impact of sociodemographic and clinical characteristics on HRQoL were compared by univariate and multivariate regression analyses.

**Results:**

In total, 285 patients responded to the survey (response rate, 65.1%). Psychological-related HRQoL variables such as emotional function (*P* = 0.021) and future perspectives (*P* = 0.044) were poorer for younger patients than for older patients; and physiological-related HRQoL was reflected by physical function (*P* = 0.039), which was poorer for older patients than for younger patients. In terms of physiologic function and symptoms concerning HRQoL, such as pain (*P* = 0.002) and insomnia (*P* = 0.018), females had lower values than males. Low education and unemployment were associated with a worse HRQoL. HRQoL was worse for patients with stomas compared to those without, especially in psychosocial areas such as role function (*P* = 0.025), social function (*P* <0.001) and body image (*P* = 0.004). Financial HRQoL was worse for younger patients and patients with stoma.

**Conclusions:**

HRQoL aspects and degrees to which they were impaired after curative surgery for rectal cancer were different when compared by many sociodemographic and clinical factors in Chinese mainland patients.

## Background

The incidence and mortality of colorectal cancer in Chinese people has been rising yearly. According to the latest report, the incidence of colorectal cancer in Chinese people was 31.39/100,000, third among malignant neoplasms; and mortality was 14.82/100,000, fifth in terms of all deaths from malignant neoplasms in China [1]. In addition, some new patterns have been observed in the population of patients with colorectal cancer in China. For example, the incidence and mortality of male patients has been shown to be higher than that of female patients, but the proportion of females has increased. Rates for urban patients were higher than those for patients from rural areas. The population with colorectal cancer also appears to be aging, a similar trend to that seen in developed countries [2].

Despite major improvements in treating rectal cancer during the last two decades, surgery is still the preferred curative treatment. As a traumatic procedure, dissection of the rectum compromises psychological as well as functional aspects of defecation, micturition and sexual function. In the past few years, survival has not been considered the single most important endpoint of studies of rectal cancer surgery; functional results after surgery, as well as health-related quality of life (HRQoL), have gained considerable prominence.

HRQoL after surgery for rectal cancer can be affected by clinical factors such as type of operation [3,4], adjuvant therapy [5,6] and complications [7]. Previous studies have also shown the association of age [8,9], sex [10,11], education [12], marital status [12,13], social support [12,14] and timing of follow-up [4,15] with HRQoL. Cultural differences can also be involved. Some recent studies evaluated the social and cultural background, geographic origin, and religion [16,17]. Studies have investigated HRQoL after rectal cancer surgery in Asian populations such as Japanese [3,18,19], Korean [20] and Chinese from Hong Kong [21]. However, because of social and cultural differences, the results from other countries may not apply to the Chinese mainland population. To our knowledge, few studies have investigated HRQoL concerning sociodemographic characteristics or societal and cultural background after rectal cancer surgery in China.

The aim of this study was to assess HRQoL outcomes among disease-free survivors after radical surgery for rectal cancer in a Chinese mainland population, and to assess the impact of sociodemographic and clinical characteristics on HRQoL.

## Methods

### Patients

Patients with rectal cancer, who underwent primary surgery at Department of Gastrointestinal Surgery, the First Affiliated Hospital of Sun Yat-sen University (Guangzhou, China) from August 2002 to February 2011, were retrospectively reviewed. Our institution is one of the main centers for gastrointestinal surgery in southern China. The inclusion criteria were: adults (aged 18 years and over), with a diagnosis of primary rectal adenocarcinoma histologically confirmed and treated with radical resection. At our center, total mesorectal excision is the usual procedure for radical surgery of rectal cancer. Exclusion criteria included follow-up of less than 6 months from the date of surgery and lack of completion of chemoradiotherapy (CRT). The 6-month time point was selected because most patients were expected to exhibit the effects of CRT and the physical and psychological conditions within 6 months of surgery [15,22]. Other exclusion criteria included: significant comorbidity during or after surgery; other major illness; local or distant recurrence or new cancers; and treatment by local excision. All patients provided written informed consent, and the study was approved by the Ethics Committee of the First Affiliated Hospital of Sun Yat-sen University (Guangzhou, China).

### Data collection

The HRQoL questionnaires were sent to patients by mail with a self-addressed and stamped return envelope, a cover letter, and a demographic questionnaire for completion. To maximize the questionnaire response rate, subjects received a telephone call reminder if they did not respond within 30 days, and a new questionnaire was sent if needed. The received questionnaires were reviewed and the subjects who returned blank items were given a telephone call, to ascertain their comprehension and acceptance of the questionnaires, and to finish the incomplete items if they agreed to respond. All aspects of the questionnaire survey and telephone interview were administered by a single person (XL).

The following data were collected from the institutional colorectal cancer database and demographic questionnaire: age; sex; occupation; marital status; education; religion; medical insurance; postoperative time (time from surgery to survey); surgery type (anterior resection (AR), abdominoperineal resection (APR), Hartmann’s procedure or coloanal anastomosis (CAA)); CRT before or after surgery; tumor, node, metastasis (TNM) stage; site of tumor (distance from the anal verge to the inferior margin); and complications and stoma. Postoperative time was defined as short-term (≤24 months), mid-term (24 to 60 months) and long-term (>60 months). Age was classified as young (<45 years old), middle age (45 to 60 years old) and old (≥60 years old) according to the age classification of World Health Organization (WHO). The sites of tumors were categorized as low (≤5 cm), middle (5 to 10 cm) and high (>10 cm) according to the distance from the inferior margin of the tumor to the anal verge.

### HRQoL instruments

HRQoL was assessed by use of the validated European Organization for Research and Treatment of Cancer (EORTC) core [23] (QLQ-C30) and colorectal cancer (QLQ-CR38) questionnaires [24]. The QLQ-C30 has 30 items, and includes five functional scales (physical, emotional, cognitive, social and role functioning), three symptom scales (fatigue, nausea/vomiting and pain), global health status and six single-item measures (dyspnea, insomnia, appetite loss, constipation, diarrhea and financial difficulties). The QLQ-CR38 has 38 items, and includes four functional scales (body image, future perspective, sexual function and sexual enjoyment) and eight symptom scales (micturition problems, gastrointestinal tract symptoms, chemotherapy side effects, defecation problems, stoma-related problems, male and female sexual problems, and weight loss).

We used simplified Chinese versions of both questionnaires, which had been translated by rigorous forward-backward translation procedures [25]. By recommended EORTC procedures [26], patient responses were converted to a scale from 0 to 100. High functional scores represented good function and high symptom scores represented more disease. For items missing within a scale, the score was calculated by using only the items for which values were available, provided at least half of the items in the scale were completed. The scales in which less than half of the items were completed were treated as missing data, which could not be analyzed.

### Statistical analysis

All data were expressed as median and range, if not otherwise specified. We compared questionnaire responders and non-responders in terms of sociodemographic and clinical variables by Pearson’s chi-square test for unpaired samples. Since data for most variables showed a skewed distribution, medians for variables were compared by the nonparametric Mann–Whitney *U*-test or the Kruskal–Wallis test. Multiple linear regression analysis was used to identify the association of sociodemographic and clinical variables and HRQoL. The HRQoL domains and variables with *P* <0.100 on univariate analysis or those considered most clinically relevant from, in part, previous HRQoL research of rectal cancer patients [27] were selected for multivariable regression models. Variables included in the models were global health status, all functioning scales of the QLQ-C30 (physical, emotional, cognitive, social and role), and symptom scales of constipation and financial difficulties, as well as body image, future perspective, sexual function and enjoyment scales, and general gastrointestinal, defecation and stoma-related scales of the QLQ-CR38.

A two-tailed *P* <0.05 was considered statistically significant. SPSS 18.0 for Windows (SPSS Inc., Chicago, IL, USA) was used for statistical analysis.

## Results

### Patients

In total, 285 (65.1%) questionnaires were returned, 16 of which were discarded for having more than 20% of blank answers according to the World Health Organization Quality of Life (WHOQOL) user manual [28]. We had 269 almost complete questionnaires with only a minority of those missing data. In these 269 questionnaires, most missing data concerned the sexuality items of QLQ-CR38: 199 (73.98%) and 187 (69.52%) patients completed items about sexual function and enjoyment, respectively, and 122 of 171 male patients (71.35%) and 20 of 98 female patients (20.41%) completed items about sexual problems. Responders and non-responders did not differ by sociodemographic and clinical characteristics (data not shown).

Characteristics of the study population are shown in Table 1. The patient age was 63 ± 12.9 years. Of the 45 patients with stomas, seven underwent a repeat colostomy because of major surgical complications after AR. Including these patients, the median time from surgery to questionnaire completion was 43 months (range 7 to 110 months).

**Table 1:** Characteristics of 269 Chinese patients who underwent radical surgery for rectal cancer

**Characteristic**	**Number (%)**
Sex	
Male	171 (63.57)
Female	98 (36.43)
Age	
Young (<45 years)	24 (8.92)
Middle age (45 to 60 years)	77 (28.62)
Old (≥60 years)	168 (62.45)
Marital status	
Married	258 (95.9)
Unmarried	11 (4.1)
Religious faith	
Yes	13 (4.8)
No	256 (95.2)
Medical insurance	
Yes	248 (92.2)
No	21 (7.8)
Education	
Primary school or less	62 (23.05)
Middle school	149 (55.39)
University or more	58 (21.56)
Occupation	
Working	120 (44.61)
Not working	149 (55.39)
TNM stage	
1	81 (30.11)
2	103 (38.29)
3	85 (31.60)
Adjuvant therapy	
Yes	124 (46.10)
No	145 (53.90)
Postoperative time	
≤24 months	72 (26.77)
24 to 60 months	100 (37.17)
>60 months	97 (36.06)
Site of tumor	
Low	69 (25.65)
Middle	135 (50.19)
High	65 (24.16)
Stoma	
Yes	45 (16.73)
No	224 (83.27)
Type of operation	
APR	35 (13.01)
AR	223 (82.90)
CAA	8 (2.97)
Hartmann’s procedure	3 (1.12)

APR, abdominoperineal resection; AR, anterior resection; CAA, coloanal anastomosis; TNM, tumor, node, metastasis.

The impact of sociodemographic characteristics on the HRQoL scores measured by QLQ-C30 and QLQ-CR38 are presented in Table 2 and Table 3, respectively. HRQoL scores were worse for younger patients compared to older patients in terms of emotional function (*P* = 0.021), future perspective (*P* = 0.044) and financial difficulties (*P* = 0.011); and were worse for older patients than for younger patients in terms of physical function (*P* = 0.039). The statistical significance of scores for future perspective by age could hardly be revealed by median and range but was obvious by age group (young, 100.1; middle age, 138.2; old, 137.8). Scores were worse for females than for males in the variables of fatigue (*P* = 0.043), pain (*P* = 0.002), insomnia (*P* = 0.018) and gastrointestinal problems (*P* = 0.029).

**Table 2:** Impact of sociodemographic characteristics on HRQoL scores measured by QLQ-C30 for patients who underwent radical surgery for rectal cancer

**Variable**	**n**	**QL**	**PF**	**EF**	**SF**	**FA**^**a**^	**PA**^**a**^	**FI**^**a**^
Age								
Young (<45 years)	24	67 (52 to 90)	90 (80 to 100)	75 (67 to 92)	75 (67 to 100)	22 (3 to 42)	8 (0 to 17)	33 (0 to 100)
Middle age (45 to 60 years)	77	75 (67 to 100)	93 (80 to 100)	92 (75 to 100)	83 (67 to 100)	11 (0 to 33)	0 (0 to 17)	33 (0 to 33)
Old (≥60 years)	168	67 (50 to 83)	87 (75 to 100)	92 (75 to 100)	100 (67 to 100)	22 (0 to 33)	0 (0 to 17)	0 (0 to 33)
*P* value		0.082	0.039	0.021	0.325	0.284	0.643	0.011
Sex								
Male	171	67 (58 to 88)	87 (80 to 100)	92 (75 to 100)	83 (67 to 100)	11 (0 to 33)	0 (0 to 17)	0 (0 to 33)
Female	98	67 (50 to 83)	87 (73 to 100)	92 (73 to 100)	100 (67 to 100)	22 (0 to 33)	0 (0 to 33)	0 (0 to 33)
*P* value		0.319	0.056	0.191	0.792	0.043	0.002	0.913
Occupation								
Working	120	75 (67 to 100)	93 (80 to 100)	92 (75 to 100)	83 (67 to 100)	11 (0 to 33)	0 (0 to 17)	33 (0 to 33)
Not working	149	67 (50 to 83)	87 (73 to 100)	92 (75 to 100)	92 (67 to 100)	22 (0 to 33)	0 (0 to 17)	0 (0 to 33)
*P* value		0.002	0.019	0.713	0.409	0.129	0.652	0.297
Education								
Primary school or less	62	67 (50 to 83)	87 (73 to 100)	92 (67 to 100)	92 (67 to 100)	22 (3 to 33)	0 (0 to 33)	33 (0 to 33)
Middle school	149	67 (67 to 83)	87 (80 to 100)	92 (83 to 100)	83 (67 to 100)	11 (0 to 33)	0 (0 to 17)	0 (0 to 33)
University or more	58	67 (50 to 85)	87 (80 to 100)	92 (75 to 100)	100 (67 to 100)	22 (0 to 44)	0 (0 to 17)	0 (0 to 33)
*P* value		0.321	0.224	0.100	0.904	0.019	0.018	0.110

Data presented as median (range). ^a^For a symptom scale/item, a high score represents a worse HRQoL. EF, emotional functioning; FA, fatigue; FI, financial difficulties; HRQoL, health-related quality of life; PA, pain; PF, physical functioning; QL, global health status; SF, social functioning.

**Table 3:** Impact of sociodemographic characteristics on HRQoL scores measured by QLQ-CR38 for patients who underwent radical surgery for rectal cancer

**Variable**	**n**	**BI**	**SEF**	**SEE**	**FU**	**GI**^**a**^	**DF**^**a,b**^	**STO**^**a,b**^
Age								
Young (<45 years)	24	89 (67 to 100)	67 (33 to 67)	67 (33 to 67)	67 (33 to 92)	7 (0 to 13)	10 (5 to 24)	43 (24 to 70)
Middle age (45 to 60 years)	77	89 (67 to 100)	33 (0 to 67)	33 (0 to 67)	67 (67 to 100)	7 (0 to 13)	10 (0 to 26)	31 (11 to 54)
Old (≥60 years)	168	78 (67 to 100)	0 (0 to 33)	0 (0 to 33)	67 (67 to 100)	0 (0 to 13)	14 (5 to 24)	33 (11 to 43)
*P* value		0.452	0.000	0.000	0.044	0.557	0.940	0.446
Sex								
Male	171	83 (67 to 100)	33 (0 to 67)	33 (0 to 67)	67 (67 to 100)	0 (0 to 13)	10 (5 to 24)	33 (14 to 43)
Female	98	89 (67 to 100)	0 (0 to 50)	0 (0 to 67)	67 (67 to 100)	7 (0 to 20)	12 (5 to 24)	29 (13 to 50)
*P* value		0.350	0.022	0.036	0.157	0.029	0.814	0.585
Occupation								
Working	120	89 (67 to 100)	33 (0 to 67)	33 (0 to 67)	67 (67 to 100)	7 (0 to 13)	12 (5 to 24)	33 (14 to 54)
Not working	149	89 (67 to 100)	0 (0 to 33)	0 (0 to 67)	67 (67 to 100)	0 (0 to 13)	10 (5 to 26)	33 (12 to 45)
*P* value		0.696	0.000	0.002	0.833	0.228	0.914	0.991
Education								
Primary school or less	62	89 (67 to 100)	0 (0 to 46)	0 (0 to 67)	67 (67 to 100)	7 (0 to 20)	14 (5 to 29)	14 (8 to 36)
Middle school	149	89 (67 to 100)	33 (0 to 67)	33 (0 to 67)	67 (67 to 100)	0 (0 to 13)	10 (5 to 24)	33 (14 to 42)
University or more	58	78 (67 to 100)	33 (0 to 67)	50 (0 to 67)	67 (67 to 100)	3 (0 to 8)	10 (5 to 24)	43 (24 to 71)
*P* value		0.425	0.042	0.018	0.539	0.225	0.677	0.048

Data presented as median (range). ^a^For a symptom scale/item, a high score represents a worse HRQoL; ^b^defecation scores concern only non-stoma patients, and stoma-related problems concern only stoma patients. BI, body image; DF, defecation problems; FU, future perspective; GI, gastrointestinal problems; HRQoL, health-related quality of life; SEE, sexual enjoyment; SEF, sexual functioning; STO, stoma-related problems.

HRQoL scores were worse for patients with lower levels compared to higher levels of education in terms of fatigue (*P* = 0.019) and pain (*P* = 0.018). Global health status scores were better for employed compared to unemployed patients (*P* = 0.002).

Scores were better for married patients than unmarried patients in terms of cognitive function (*P* = 0.012), and symptoms such as fatigue (*P* <0.001), insomnia (*P* = 0.004) and gastrointestinal symptoms (*P* = 0.005). There were no differences in future perspectives (*P* = 0.626) between religious and non-religious (based on a ‘yes’ or ‘no’ questionnaire response) patients. Patients with and without medical insurance did not differ in terms of financial difficulty scores (*P* = 0.109).

The scores on sexual function and sexual enjoyment were better for younger patients compared to older patients (*P* <0.001, <0.001), males compared to females (*P* = 0.022, 0.036), employed compared to unemployed patients (*P* <0.001, 0.002) and patients with high compared to low education (*P* = 0.042, 0.018).

The impact of clinical characteristics on HRQoL scores measured by QLQ-C30 and QLQ-CR38 are shown in Table 4 and Table 5, respectively. Compared to patients with stomas, patients without stomas had better HRQoL scores for physical function (*P* = 0.034), role function (*P* = 0.025), emotional function (*P* = 0.011), social function (*P* <0.001), financial difficulties (*P* <0.001), body image (*P* = 0.004), future perspective (*P* = 0.049) and weight loss (*P* = 0.027). Only for constipation were scores better for patients with stomas compared to those without stomas (*P* = 0.004).

**Table 4:** Impact of clinical characteristics on HRQoL scores measured by QLQ-C30 for patients who underwent radical surgery for rectal cancer

**Variable**	**n**	**QL**	**PF**	**EF**	**SF**	**FA**^**a**^	**PA**^**a**^	**FI**^**a**^
Stoma								
Yes	45	67 (50 to 83)	87 (73 to 93)	83 (67 to 100)	67 (67 to 83)	11 (0 to 33)	0 (0 to 33)	33 (17 to 67)
No	224	67 (56 to 83)	87 (80 to 100)	92 (75 to 100)	100 (67 to 100)	22 (0 to 33)	0 (0 to 17)	0 (0 to 33)
*P* value		0.462	0.034	0.011	0.000	0.645	0.111	0.000
Type of operation								
APR	35	67 (50 to 83)	87 (73 to 93)	83 (67 to 100)	67 (67 to 83)	11 (0 to 33)	17 (0 to 33)	33 (0 to 33)
AR	223	67 (54 to 83)	87 (80 to 100)	92 (75 to 100)	100 (67 to 100)	22 (0 to 33)	0 (0 to 17)	0 (0 to 33)
CAA	8	88 (42 to 100)	87 (80 to 98)	96 (77 to 100)	83 (67 to 100)	11 (0 to 31)	0 (0 to 13)	17 (0 to 58)
Hartmann’s procedure	3	63 (58 to 67)	67 (67 to 73)	83 (33 to 100)	17 (0 to 83)	33 (11 to 33)	0 (0 to 50)	100 (67 to 100)
*P* value		0.687	0.073	0.204	0.000	0.735	0.332	0.000
Postoperative time								
≤24 months	72	67 (58 to 83)	87 (75 to 100)	92 (67 to 100)	83 (67 to 100)	22 (0 to 33)	0 (0 to 33)	33 (0 to 33)
24 to 60 months	100	67 (50 to 83)	87 (75 to 100)	92 (75 to 100)	83 (67 to 100)	22 (0 to 33)	0 (0 to 17)	0 (0 to 33)
>60 months	97	67 (58 to 83)	87 (80 to 100)	92 (79 to 100)	100 (67 to 100)	11 (0 to 28)	0 (0 to 17)	0 (0 to 33)
*P* value		0.993	0.798	0.160	0.070	0.358	0.156	0.038
Site of tumor								
Low	69	67 (50 to 83)	87 (80 to 100)	92 (75 to 100)	67 (67 to 100)	11 (0 to 33)	0 (0 to 17)	33 (0 to 33)
Middle	135	67 (50 to 83)	87 (80 to 100)	92 (75 to 100)	83 (67 to 100)	22 (0 to 33)	0 (0 to 17)	0 (0 to 33)
High	65	67 (67 to 83)	93 (76 to 100)	92 (75 to 100)	100 (83 to 100)	11 (0 to 33)	0 (0 to 17)	0 (0 to 33)
*P* value		0.631	0.574	0.839	0.001	0.420	0.287	0.095

Data presented as median (range). ^a^For a symptom scale/item, a high score represents a worse HRQoL. APR, abdominoperineal resection; AR, anterior resection; CAA, coloanal anastomosis; EF, emotional functioning; FA, fatigue; FI, financial difficulties; HRQoL, health-related quality of life; PA, pain; PF, physical functioning; QL, global health status; SF, social functioning.

**Table 5:** Impact of clinical characteristics on HRQoL scores measured by QLQ-CR38 for patients who underwent radical surgery for rectal cancer

**Variable**	**n**	**BI**	**SEF**	**SEE**	**FU**	**GI**^**a**^	**DF**^**a,b**^	**STO**^**a,b**^
Stoma								
Yes	45	67 (56 to 97)	0 (0 to 83)	33 (0 to 67)	67 (67 to 100)	7 (0 to 13)	-	33 (14 to 45)
No	224	89 (67 to 100)	33 (0 to 100)	33 (0 to 67)	67 (67 to 100)	0 (0 to 13)	10 (5 to 24)	-
*P* value		0.004	0.396	0.427	0.049	0.400	-	-
Type of operation								
APR	35	67 (56 to 100)	0 (0 to 50)	33 (0 to 67)	67 (67 to 100)	7 (0 to 13)	-	33 (14 to 48)
AR	223	89 (67 to 100)	25 (0 to 67)	33 (0 to 67)	67 (67 to 100)	0 (0 to 13)	10 (5 to 24)	19 (0 to 71)
CAA	8	94 (81 to 100)	58 (13 to 79)	67 (17 to 92)	67 (67 to 100)	0 (0 to 0)	12 (6 to 26)	-
Hartmann’s procedure	3	78 (56 to 100)	8 (0 to 17)	NA	67 (0 to 100)	0 (0 to 7)	-	38 (29 to 43)
*P* value		0.126	0.415	0.206	0.391	0.227	0.836	0.653
Postoperative time								
≤24 months	72	89 (67 to 100)	17 (0 to 50)	33 (0 to 67)	67 (67 to 100)	0 (0 to 13)	24 (7 to 36)	33 (14 to 43)
24 to 60 months	100	89 (67 to 100)	33 (0 to 67)	33 (0 to 67)	67 (67 to 100)	0 (0 to 7)	12 (5 to 24)	24 (14 to 38)
>60 months	97	89 (67 to 100)	17 (0 to 67)	33 (0 to 67)	67 (67 to 100)	7 (0 to 13)	10 (0 to 19)	33 (10 to 67)
*P* value		0.951	0.993	0.972	0.763	0.414	0.000	0.685
Site of tumor								
Low	69	78 (67 to 100)	0 (0 to 50)	33 (0 to 67)	67 (67 to 100)	7 (0 to 13)	17 (10 to 29)	33 (14 to 50)
Middle	135	89 (67 to 100)	25 (0 to 67)	33 (0 to 67)	67 (67 to 100)	0 (0 to 13)	14 (5 to 29)	17 (11 to 36)
High	65	89 (67 to 100)	33 (0 to 67)	33 (0 to 67)	67 (67 to 100)	0 (0 to 20)	5 (0 to 19)	29 (0 to 76)
*P* value		0.332	0.621	0.587	0.504	0.731	0.010	0.395

Data presented as median (range). ^a^For a symptom scale/item, a high score represents a worse HRQoL; ^b^defecation scores concern only non-stoma patients, and stoma-related problems concern only stoma patients. BI, body image; DF, defecation problems; FU, future perspective; GI, gastrointestinal problems; HRQoL, health-related quality of life; SEE, sexual enjoyment; SEF, sexual functioning; STO, stoma-related problems.

When different types of surgical procedures were considered, AR patients had the best social function (*P* <0.001), and most commonly had constipation (*P* = 0.004). Both APR and Hartmann’s procedure patients with stomas had greater concerns about financial difficulties (*P* <0.001). Scores for patients with low rectal cancer were poorer for male sexual problems (*P* = 0.032), social function (*P* = 0.001) and defecation problems (*P* = 0.010). Scores were better for long-term than mid-term and short-term postoperative time in terms of financial difficulties (*P* = 0.038) and defecation problems (*P* <0.001).

The factors predicting HRQoL scores are presented in Table 6. The presence of stomas was the most significant negative characteristic predicting scores in seven HRQoL areas: emotional function (*P* = 0.019), social function (*P* = 0.003), financial difficulties (*P* = 0.001), body image (*P* <0.001), future perspective (*P* = 0.047), sexual function (*P* = 0.049) and sexual enjoyment (*P* = 0.028) (scores for sexual areas not shown). Furthermore, it was the only independent predictor of future perspective HRQoL. Young age, increased postoperative months and employment were independently associated with high HRQoL for physical function (*P* = 0.001), role function (*P* = 0.038) and global health status (*P* = 0.009), respectively. Factors associated with poor financial HRQoL were young patients (*P* = 0.002), low education (*P* = 0.003), fewer postoperative months (*P* = 0.008) and the presence of stomas (*P* = 0.001) (Additional file 1: Tables S1, S2 and S3).

**Table 6:** Multiple linear regression analysis of sociodemographic and clinical variables significantly associated with HRQoL scores for patients who underwent radical surgery for rectal cancer

**Variable**	**PF**	**RF**	**EF**	**SF**	**QL**	**FI**^**a**^	**BI**	**FU**
	**(n = 269)**	**(n = 269)**	**(n = 269)**	**(n = 268)**	**(n = 265)**	**(n = 268)**	**(n = 266)**	**(n = 268)**
Age	−0.196^b^	−0.067	0.076	0.052	−0.050	−0.201^b^	−0.073	0.119
Sex	−0.119	−0.066	−0.071	0.043	−0.032	-	0.009	−0.127
Occupation	-	0.046	-	0.015	0.178^c^	−0.046	−0.014	0.050
Education	-	-	0.013	0.078	-	−0.174^b^	−0.049	−0.038
Stoma	−0.106	−0.092	−0.144^b^	−0.197^b^	−0.064	0.209^b^	−0.239^b^	−0.122^b^
TNM stage	−0.088	−0.053	−0.123^b^	−0.091	-	-	-	-
Postoperative months	0.054	0.128^b^	-	0.125^b^	0.021	−0.157^b^	0.005	0.018
Site of tumor	-	-	-	0.105	-	−0.001	-	-

Data presented as beta values. ^a^For a symptom scale/item, a high score represents worse HRQoL; ^b^*P* <0.05. BI, body image; CF, cognitive functioning; EF, emotional functioning; FI, financial difficulties; FU, future perspective; HRQoL, health-related quality of life; PF, physical functioning; QL, global health status; RF, role functioning; SF, social functioning; TNM, tumor, node, metastasis.

## Discussion

We investigated HRQoL in Chinese patients who underwent curative surgery for rectal cancer. Our results indicate that the HRQoL aspects and the degrees to which they were impaired after curative surgery for rectal cancer were different when compared by many sociodemographic and clinical factors in Chinese mainland patients. Many studies have demonstrated that HRQoL varies by culture, social background and geographic origin [16,17,20]. China has a unique history, traditional culture, ideology and social security system. The population of the current study consisted largely of older people and retired workers who were conservative and traditional thinkers, with unique education, employment, medical insurance and religious beliefs. Their values and views of disease and treatment differ considerably from those in Western countries and even other countries in Asia. These factors may have a great impact on HRQoL in patients who underwent radical surgery for rectal cancer.

### Impact of sociodemographic characteristics on HRQoL

In line with many previous findings [10,11,19,27], female patients were more affected than males in physiologic function and symptoms concerning HRQoL. Interestingly, our results overlap with some results even in the same scales as insomnia, fatigue and sexual function [10]. Kroenke and Spitzer [29] had attributed the reasons for sex differences in symptom reporting to psychosocial and cultural factors, social roles and responsibilities, and physiologic differences between males and females. Our data clearly indicated that there were no differences in this respect between Eastern and Western women. As Krouse *et al*. [11] reported, women were found to engage in more coping behavior and sought more social support than men, including emotional and spiritual activities.

Sideris *et al*. [10] found that patients with middle and low levels of education, and the unemployed had worse scores in some symptom HRQoL scales compared to their counterparts. We found similar results. On multivariate regression analysis, low education was significantly and independently associated with low financial HRQoL, and unemployment was the only independent and significant predictor of worse global health status. Additional social support may help patients in poor social environments with HRQoL after surgery.

Family and marriage may provide more emotional care and financial support to patients in distress. We found better scores for married patients compared to unmarried patients in many HRQoL scales. People in the East place more importance on family than those in the West. Moreover, compared with other studies concerning marital status, we found that the marriage rate of Chinese (more than 95%) was obviously higher than people of European descent (less than 80%) [10,30]. Following radical surgery for rectal cancer, Chinese patients may benefit in HRQoL from a contented married life and high marriage rate. In the current study, the proportion of people who checked the ‘religious’ box on the questionnaire was low, and we found no differences in some religion-related scales, such as future perspective between religious and non-religious patients. For the different national conditions and social characteristics, the marriage and religion situations of China are quite different from other countries. Although few studies have exclusively focused on the impact of religion and marriage on HRQoL after surgery for rectal cancer, these two characteristics were thought to be patient-related factors impacting changes in quality of life [10,17].

In the current study, the percentages of completion of scales for male and female sexual problems were only 71.35% and 20.41%, respectively. The scores for sex-related scales were relatively low, but could not be compared with HRQoL normative data for an Asian population, which are not available. This result suggested that the sexual function of these patients was more affected by surgery. One study from Hong Kong [21], the most westernized city of China, found that ratings for sexual function and enjoyment were lower than those in Western countries. The authors concluded that the Chinese culture generally puts less emphasis on sexuality and views intimacy as independent of sexual relations. The current study population consisted of mostly older people with low education, which may have contributed to the low response rates and low scores in sex-related scales. Many studies involving the same edition of the EORTC QLQ-CR38 in different countries and districts, such as Hong Kong [21], Japan [3], Italy [27] and Switzerland [31], found low response rates in scales for sexual function, enjoyment and problems, especially for females. Sex-related investigation in studies of HRQoL may always be difficult, regardless of culture, especially for females. On the other hand, in the current study, some patients reported that the sex-related items were difficult to understand and answer; therefore, the value of the sex-related items in the EORTC QLQ-CR38 is questionable, which is similar to the findings in a previous study [32].

### Impact of clinical characteristics on HRQoL

Similar to other studies [3,10], HRQoL after rectal cancer surgery was worse in the early postoperative period in terms of financial difficulties and defecation problems, but improved with time after surgery. From multivariate regression analysis, it was shown that an increase in postoperative period was associated with high scores for role function, social function and financial status. Scores were largely similar by age group 5 years after surgery. HRQoL were more impaired for patients with low compared to high rectal cancers, for male sexual problems, social function and defecation, which is in agreement with other studies [4,17].

Scores were worse for patients with stomas compared to those without stomas in 9 of the 27 HRQoL scales. The differences lay mostly in the functioning scales (6 of 9), especially those closely related to psychological and social aspects, namely role, emotional and social functions, as well as body image and future perspectives. On multivariate analysis, the presence of stomas was the best predictor of HRQoL. Patients with stomas, especially older Chinese patients, might be important in maintaining an intact body all the way to the end of life. More psychological counseling may be needed [16] to improve HRQoL of patients with stomas. However, patients without stomas still showed poor HRQoL for constipation, which paralleled those of AR patients. Pachler and Wille-Jorgensen [33] could not draw any definite conclusions about the relation of stomas and HRQoL after an exhaustive review of the literature. Our scores for constipation in patients with stomas and those for psychosocial function in patients without stomas were better than their counterparts. Similar results were observed in prospective or retrospective studies by Engel *et al*. [4], Guren *et al*. [34], Camilleri-Brennan J *et al*. [35] and Ross *et al*. [36].

### Finance-related HRQoL

HRQoL scores dealing with finance were better for older than for middle aged and younger patients. From the multivariate analysis, besides young age and presence of stomas, less education and shorter postoperative time independently predicted poor financial HRQoL. The medical security policy of China consists of basic, complementary, commercial medical insurance and social medical aid. This policy covers a broad population, and only 7.8% of our patients had no medical insurance. Under this policy, older patients can share in the financial security with their retirement pension and medical insurance. In addition, in Chinese culture, children support their parents when they are old. Most children can afford the high medical expenditures for their parents, thus older patients are not so worried about financial problems. However, middle aged and young patients are the economic backbone for their immediate family and their parents. Therefore, patients from countries with more well-established social welfare policies may have lower financial burdens after their illness and surgery [10], which can explain the association of young age and low HRQoL in terms of financial status.

In addition, patients with stomas have additional financial difficulties such as the cost of the devices and materials each month. In China, no charges regarding stoma apparatus are covered by outpatient medical insurance. Kuzu *et al*. [17] found that patients in poor areas might have problems managing stomas because of the lack of proper supportive care. Even in well-developed countries, having low income and problems paying for stoma supplies can affect patient HRQoL [10]. Medical insurance coverage of the cost of stoma devices and materials may help to improve the HRQoL of patients with stomas.

### Limitations

The response rate to our mailed questionnaire was 65.1%, which is a little higher than the lower range for similar studies of rectal cancer patients (54 to 81%) [4,11,19,37]. The reasons for the low response rate may be the older age and middle- to low-level of education of our sample, the extended postoperative time, and the use of mailed questionnaires as the major investigation method. However, greater than 50% response rate is considered adequate for a mailed survey study [38], and the present sample may represent the Chinese mainland population because the sample size was reasonably large. Furthermore, data for some subgroups with small numbers, such as patients who were unmarried, with no medical insurance and who were religious, might have obscured significant differences, and resulted in the possibility of type 1 error and a lack of persuasiveness. We could not compare our data to the HRQoL data to general Asian populations because such data are lacking. At present, only two studies involving use of the EORTC QLQ-C30 questionnaire in German [39] and Norwegian [40] general populations are available. Finally, we had expected some different quality of life results in rectal cancer patients between Eastern and Western populations. The lack of differences in the quality of life data between the populations was rather surprising. The reasons for this have not been determined, but will be addressed in future work.

## Conclusions

Our results indicate that the HRQoL aspects and degrees to which they are impaired after curative surgery for rectal cancer were different when compared by many sociodemographic and clinical factors in Chinese mainland patients. Psychological counseling might help younger patients undergoing rectal surgery. For older patients and females, more emphasis should be placed on body function recovery. Additional social support could help patients in poor social environments improve HRQoL after surgery. The presence of stomas had a negative impact on HRQoL, mainly psychosocial and financial HRQoL. Therapy, follow-up, research and social support during postoperative recovery should be more targeted. When these results are compared to studies from Western countries, cultural differences did not appear to influence HRQoL.

## Abbreviations

APR: Abdominoperineal resection; AR: Anterior resection; CAA: Coloanal anastomosis; CRT: Chemoradiotherapy; EORTC: European Organization for Research and Treatment of Cancer; HRQoL: Health-related quality of life; TNM: Tumor node, metastasis; WHO: World Health Organization; WHOQOL: World Health Organization Quality of Life.

## Competing interests

The authors declare that they have no competing interests.

## Authors’ contributions

XS and XL conceived the study and design. XL, ZC, ML and LL undertook acquisition of data. XL, KX and YX performed analysis and interpretation of data. XL drafted the manuscript. XS, WZ and YH critically reviewed the manuscript. All authors read and approved the final manuscript.

## Supplementary Material

Additional file 1: Table S1 Impact of Sociodemographic and Clinical Characteristics on RF and CF Scales for Patients Who Underwent Radical Surgery for Rectal Cancer. **Table S2.** Impact of Sociodemographic Characteristics on Partial HRQoL Scores for Patients Who Underwent Radical Surgery for Rectal Cancer. **Table S3.** Impact of Clinical Characteristics on Partial HRQoL Scores for Patients Who Underwent Radical Surgery for Rectal Cancer. Click here for file
