# Supplementary material for: Quality of life in rectal cancer patients after radical surgery: a survey of Chinese patients
Source: World J Surg Oncol. 2014 May 22;12:161. doi: 10.1186/1477-7819-12-161 (PMC4059026; doi:10.1186/1477-7819-12-161)
Supplement: Additional file 1: Table S1 — Impact of Sociodemographic and Clinical Characteristics on RF and CF Scales for Patients Who Underwent Radical Surgery for Rectal Cancer. Table S2. Impact of Sociodemographic Characteristics on Partial HRQoL Scores for Patients Who Underwent Radical Surgery for Rectal Cancer. Table S3. Impact of Clinical Characteristics on Partial HRQoL Scores for Patients Who Underwent Radical Surgery for Rectal Cancer. [file 1477-7819-12-161-S1.doc]

| Table S1. Impact of Sociodemographic and Clinical Characteristics on RF and CF Scales for Patients Who Underwent Radical Surgery for Rectal Cancer | | | | | | | | |
| --- | --- | --- | --- | --- | --- | --- | --- | --- |
| Variable | N | RF | CF |  | Variable | N | RF | CF |
| Age |  |  |  |  | Stoma |  |  |  |
| Youth | 24 | 100(67-100) | 83(50-100) |  | Yes | 45 | 100(67-100) | 83(50-83) |
| Middle Age | 77 | 100(100-100) | 83(67-100) |  | No | 224 | 100(100-100) | 83(67-100) |
| Old Age | 168 | 100(83-100) | 83(50-100) |  | *P value* |  | 0.025 | 0.352 |
| *P value* |  | 0.111 | 0.090 |  | Type of Operation |  |  |  |
| Sex |  |  |  |  | APR | 35 | 100(67-100) | 83(50-83) |
| Male | 171 | 100(100-100) | 83(67-100) |  | AR | 223 | 100(100-100) | 83(67-100) |
| Female | 98 | 100(83-100) | 83(50-100) |  | CAA | 8 | 100(83-100) | 83(58-96) |
| *P value* |  | 0.439 | 0.185 |  | Hartmann | 3 | 83(50-100) | 83(67-100) |
| Occupation |  |  |  |  | *P value* |  | 0.145 | 0.652 |
| Working | 120 | 100(100-100) | 83(67-100) |  | Postoperative Months |  |  |  |
| Not Working | 149 | 100(83-100) | 83(58-100) |  | ≤24 | 72 | 100(83-100) | 83(67-100) |
| *P value* |  | 0.209 | 0.199 |  | 24–60 | 100 | 100(83-100) | 83(54-100) |
| Education |  |  |  |  | ＞60 | 97 | 100(100-100) | 83(58-92) |
| Primary School or Less | 62 | 100(83-100) | 75(50-88) |  | *P value* |  | 0.072 | 0.225 |
| Middle School | 149 | 100(100-100) | 83(67-100) |  | Site of Tumor |  |  |  |
| University or More | 58 | 100(83-100) | 83(67-100) |  | Low | 69 | 100(83-100) | 83(50-83) |
| *P value* |  | 0.169 | 0.373 |  | Middle | 135 | 100(83-100) | 83(67-100) |
|  |  |  |  |  | High | 65 | 100(100-100) | 83(67-100) |
|  |  |  |  |  | *P value* |  | 0.403 | 0.620 |
| Data present as median (range). RF: role functioning; CF: cognitive functioning | | | | | | | | |

| Table S2. Impact of Sociodemographic Characteristics on Partial HRQoL Scores for Patients Who Underwent Radical Surgery for Rectal Cancer | | | |
| --- | --- | --- | --- |
| Variable | N | Scale | |
| Sex |  | SL^a^ | GI^a^ |
| Male | 171 | 0(0-33) | 0(0-13) |
| Female | 98 | 0(0-33) | 20(7-27) |
| *P value* |  | 0.018 | 0.029 |
|  |  |  |  |
| Marital Status |  | CF | FA^a^ |
| Married | 258 | 83(67-100) | 22(0-33) |
| Unmarried | 11 | 50(33-83) | 56(33-67) |
| *P value* |  | 0.012 | 0.000 |
|  |  |  |  |
| Marital Status |  | SL^a^ | GI^a^ |
| Married | 258 | 0(0-33) | 0(0-13) |
| Unmarried | 11 | 33(0-67) | 20(7-27) |
| *P value* |  | 0.004 | 0.005 |
|  |  |  |  |
| Religion |  | FU |  |
| Yes | 13 | 67(50-100) |  |
| No | 256 | 67(67-100) |  |
| *P value* |  | 0.626 |  |
|  |  |  |  |
| Medical Insurance |  | FI^a^ |  |
| Yes | 248 | 0(0-33) |  |
| No | 21 | 33(0-67) |  |
| *P value* |  | 0.109 |  |
| Data present as median (range).  a For a symptom scale/item, a high score represents worse HRQoL.  SL: insomnia; GI: symptoms in the area of the gastrointestinal tract;  CF: cognitive functioning; FA: fatigue;  FU: future perspective; FI: financial difficulties | | | |

| Table S3. Impact of Clinical Characteristics on Partial HRQoL Scores for Patients Who Underwent Radical Surgery for Rectal Cancer | | | |
| --- | --- | --- | --- |
| Variable | N | Scale | |
| Stoma |  | CO^a^ | WL^a^ |
| Yes | 45 | 0(0-33) | 0(0-17) |
| No | 224 | 33(0-33) | 0(0-0) |
| *P value* |  | 0.004 | 0.027 |
|  |  |  |  |
| Type of Operation |  | CO^a^ |  |
| APR | 35 | 0(0-33) |  |
| AR | 223 | 33(0-33) |  |
| CAA | 8 | 0(0-33) |  |
| Hartmann | 3 | 0(0-50) |  |
| *P value* |  | 0.004 |  |
|  |  |  |  |
| Site of Tumor |  | DF^a^ | MSX^a^ |
| Low | 69 | 17(10-29) | 33(17-67) |
| Middle | 135 | 14(5-29) | 42(0-88) |
| High | 65 | 5(0-19) | 17(0-33) |
| *P value* |  | 0.010 | 0.032 |
|  |  |  |  |
| Postoperative Months |  | DF^a^ |  |
| ≤24 | 72 | 24(7-36) |  |
| 24–60 | 100 | 12(5-24) |  |
| ＞60 | 97 | 10(0-19) |  |
| *P value* |  | 0.000 |  |
| Data present as median (range).  a For a symptom scale/item, a high score represents worse HRQoL.  CO: constipation; WL: weight lose;  DF: defecation problems; MSX: male sexual problems | | | |
